# Hepatocytic Differentiation Potential of Human Fetal Liver Mesenchymal Stem Cells:* In Vitro* and* In Vivo* Evaluation

**DOI:** 10.1155/2016/6323486

**Published:** 2016-01-13

**Authors:** Hoda El-Kehdy, Guillaume Pourcher, Wenwei Zhang, Zahia Hamidouche, Sylvie Goulinet-Mainot, Etienne Sokal, Pierre Charbord, Mustapha Najimi, Anne Dubart-Kupperschmitt

**Affiliations:** ^1^Inserm U972, Paul Brousse Hospital, 94807 Villejuif, France; ^2^UMR_S972, Université Paris-Sud, 94807 Villejuif, France; ^3^Département Hospitalo-Universitaire Hepatinov, Hôpital Paul Brousse, 94807 Villejuif, France; ^4^Département de Chirurgie Digestive Minimale-Invasive, Hôpital Antoine Béclère, APHP, 92141 Clamart, France; ^5^Université Catholique de Louvain, Institut de Recherche Expérimentale and Clinique (IREC), Laboratory of Pediatric Hepatology and Cell Therapy, 1200 Brussels, Belgium; ^6^Pierre and Marie Curie University, IBPS Laboratory of Developmental Biology, UMR CNRS 7622, INSERM U1156, 75005 Paris, France

## Abstract

In line with the search of effective stem cell population that would progress liver cell therapy and because the rate and differentiation potential of mesenchymal stem cells (MSC) decreases with age, the current study investigates the hepatogenic differentiation potential of human fetal liver MSCs (FL-MSCs). After isolation from 11-12 gestational weeks' human fetal livers, FL-MSCs were shown to express characteristic markers such as CD73, CD90, and CD146 and to display adipocytic and osteoblastic differentiation potential. Thereafter, we explored their hepatocytic differentiation potential using the hepatogenic protocol applied for adult human liver mesenchymal cells. FL-MSCs differentiated in this way displayed significant features of hepatocyte-like cells as demonstrated* in vitro* by the upregulated expression of specific hepatocytic markers and the induction of metabolic functions including CYP3A4 activity, indocyanine green uptake/release, and glucose 6-phosphatase activity. Following transplantation, naive and differentiated FL-MSC were engrafted into the hepatic parenchyma of newborn immunodeficient mice and differentiated* in situ*. Hence, FL-MSCs appeared to be interesting candidates to investigate the liver development at the mesenchymal compartment level. Standardization of their isolation, expansion, and differentiation may also support their use for liver cell-based therapy development.

## 1. Introduction

Human mesenchymal stem cells (MSC) are gaining more and more interest for cell therapy purposes because of their stemness (capacity for self-renewal and multipotentiality) and their property to secrete a number of antiapoptotic and anti-inflammatory factors essential for tissue repair [1]. These features support their clinical applicability for autologous and, eventually, allogeneic transplantation.

It is now accepted that MSCs are present, both in humans and in mice, not only in the bone marrow (BM), but also in many adult and fetal tissues [2, 3]. However, MSCs from different origins may present specific characteristics and properties. In particular, it has been shown that human fetal liver (FL) MSCs display specific characteristics as compared to those of BM, that is, lesser immune suppressive capacity, larger expansion potential, longer telomeres, and higher telomerase activity [4–6]. Such differences may be related to a distinct gene profile in FL-MSCs [7]. Moreover, we have recently observed that the differentiation default pathway was myogenic in FL-MSCs, contrarily to that of BM-MSCs known to be osteogenic [8].

In this work, we further explore the specificity of FL-MSCs, making the hypothesis that the site of origin of these cells would imprint on their characteristics. We show that FL-MSCs, cultured in conditions favoring the generation of hepatocyte-like cells from mesenchymal cells of human adult livers [9], could also differentiate into functional hepatocyte-like cells. Furthermore, 8 weeks after transplantation in the liver of newborn immunodeficient mice, both nondifferentiated and differentiated FL-MSCs gave rise to foci of hepatocytes, showing that they are able to further differentiate* in vivo* into mature hepatocytes.

## 2. Materials and Methods

### 2.1. Human Fetal Liver Mesenchymal Stem Cell Isolation

Human fetal livers were carefully dissected from fetuses collected after voluntary pregnancy termination at gestational weeks (GW) 11-12 with the written mothers' consent, abiding by the ethical French guidelines and the amended Declaration of Helsinki. Fetal livers were incubated under slow agitation on a heating magnetic stirrer for 1 hour at 37°C with an enzyme mixture solution containing hyaluronidase (Sigma) and collagenases I and IV (Sigma). Recovered cells were suspended in 10 mL of expansion medium *α*MEM (Gibco) supplemented with 10% fetal calf serum (Thermo Scientific HYCLONE), 2 mM glutamine (Gibco), 100 U/mL penicillin/streptomycin (Gibco), and 1 ng/mL FGF2 (R&D Systems). Cell suspension was then filtered using a 70 *μ*m cell strainer (Falcon), centrifuged and washed three times using expansion medium at 50 g for 5 min at room temperature. Cell pellets were suspended in expansion medium and seeded at density of 5.2 × 10^4^/cm^2^ on plastic culture flasks [8] and incubated at 37°C in fully humidified atmosphere containing 5% CO_2_. After 24 h, medium was completely changed to eliminate nonadherent hematopoietic cells (95% of cell population) and thereafter renewed every 3 days. At confluence, cells were detached with 0.25% w/v trypsin-EDTA for 3 min (Gibco) and seeded at density of 2.1 × 10^4^ cells/cm^2^. Cells from passages 3 to 5 were used for the characterization studies.

### 2.2. Flow Cytometry

FL-MSCs were harvested with trypsin-EDTA, centrifuged at 1200 rpm for 5 min, and suspended in PBS supplemented with 0.1% bovine serum albumin (BSA). Cell aliquots (2 × 10^4^ cells) were incubated for 20 min at 4°C with conjugated specific antibodies or corresponding isotypes (Table 1). Cells were then washed and suspended for analysis using Accuri C6 flow cytometer and CyFlow software (BD Biosciences).

### 2.3. Immunofluorescence

FL-MSCs grown on collagen type I coated labtek were fixed with 4% paraformaldehyde (Sigma) for 15 min at room temperature and permeabilized with 0.1% Triton X-100 (Sigma) for 10 min. Nonspecific immunostaining was prevented by 30 min incubation in PBS with 3% (w/v) BSA. Cells were incubated for 1 hour with primary antibodies at room temperature (see Table 1). After washing step, cells were incubated for 30 min with secondary antibodies at room temperature (Table 1). Nuclei were stained for 5 min with DAPI (Life Technologies). Slides were mounted in fluoromount medium (Sigma). Fluorescence was assessed using Imager A1 fluorescent microscope (Carl Zeiss) and digital images were acquired using Axiovision Software.

### 2.4. *In Vitro* Mesenchymal Differentiation

To induce adipogenic differentiation, FL-MSCs were seeded at 20 000 cells/cm^2^ and treated with adipogenic medium for 21 days with medium change 3 times weekly. Adipogenic medium consists of DMEM-high glucose (4,5 g/L) (Gibco) supplemented with 10% fetal calf serum, 1 *μ*M dexamethasone (Sigma), 0,5 mM isobutyl-methylxanthine (IBMX) (Sigma), 60 *μ*M indomethacin (Sigma), 1 *μ*g/L insulin (Sigma), and 10 *μ*M rosiglitazone (Santa Cruz). Adipogenesis was assessed by oil red O staining (Sigma) after 21 days.

To induce osteogenic differentiation, cells were seeded at 15 000 cells/cm^2^ treated with osteogenic medium for 21 days with medium change 3 times weekly. Osteogenic medium consists of DMEM-high glucose with 10% fetal calf serum, 0.1 *μ*M dexamethasone (Sigma), 25 *μ*g/mL L-ascorbic acid (Sigma), and 3 mM NaH_2_PO_4_ (Sigma). Osteogenesis was assessed by alkaline phosphatase activity (Sigma) and alizarin red (Sigma) staining after 6 and 21 days, respectively.

### 2.5. *In Vitro* Hepatocytic Differentiation

Hepatocytic differentiation was induced as described previously with minor modifications [9]. FL-MSCs were seeded at a density of 1 × 10^4^/cm^2^ on rat tail collagen type I (BD) coated flasks (Falcon) using expansion medium. After 24 hours, cells were cultured in serum-free IMDM medium (Gibco) supplemented with 20 ng/mL epidermal growth factor (R&D Systems) and 10 ng/mL FGF2 (Peprotech). Forty-eight hours later, cell differentiation was induced after incubation with IMDM containing 20 ng/mL hepatocyte growth factor (Peprotech), 10 ng/mL FGF2, and 0,61 g/L nicotinamide (Sigma). Seven days later, cell maturation was induced using IMDM containing 20 ng/mL oncostatin M (Peprotech), 1 *μ*M dexamethasone (Sigma), and 50 mg/mL ITS premix (BD). For the last two steps, the medium was changed every 3 days. At the end of each step, mRNA expression level was studied. At the end of the whole differentiation process, quality of hepatogenic differentiation was concomitantly evaluated at the morphology, phenotype, and functional levels.

Undifferentiated cells using both in* in vitro* and* in vivo* studies represent FL-MSC seeded and cultured in the same conditions using IMDM containing 2% FCS and 1% P/S and with no growth factor or cytokine.

### 2.6. Uptake of Low Density Lipoprotein (LDL)

Differentiated and nondifferentiated FL-MSCs were incubated with 5 *μ*g/mL Dil (1,10-dioctadecyl-1′-3,3,3′,3′-tetramethyl-indo-carbocyaninperchlorate) conjugated to LDL (Biomedical Technologies) for 3 hours at 37°C and then washed 3 times with serum-free medium and 3 times with PBS 1x. Thereafter, cells were fixed with 3% paraformaldehyde for 2 min and mounted with fluoromount medium. Red fluorescence was assessed using Imager A1 microscope (Carl Zeiss) and digital images were acquired using Axiovision Software.

### 2.7. Glucose-6-Phosphatase (G6Pase) Activity Assay

Cells were washed 2 times with PBS then incubated in 2 mL of buffer 0.1 M Tris-acetate, pH 6.5, containing 2.08 mM glucose-6-phosphate and 2.4 mM nitric lead at 37°C in humidified atmosphere containing 5% CO_2_. Glucose-6-phosphate is in these conditions transformed into glucose and the nitrate will precipitate. Four hours later, supernatant was eliminated and cells were washed for 10 sec in 1 mL of 5% ammonium sulfide to convert lead nitrate into brownish lead sulfate. Cells were examined using HP50 inverted microscope coupled to a DFC camera (Leica, Switzerland). Digital images were acquired using Leica IM50 Image Manager Software.

### 2.8. Indocyanine Green (ICG) Uptake and Release

Indocyanine green is a nontoxic organic anion exclusively eliminated by hepatocytes through the LTS1 (Liver-Specific Organic Transporter-1). For detection of cellular uptake of ICG, differentiated and undifferentiated FL-MSCs were incubated for 1 hour with 1 mg/mL ICG (Sigma) at 37°C. ICG was cleared after overnight incubation. The uptake and the clearance of ICG were assessed using a white light inverted microscope.

### 2.9. Cytochrome P450 3A4 Metabolic Activity Assay

We used the P450-Glo CYP3A4 assay where the measurement of luciferase reports the P450 cytochrome activity (Promega). Briefly, FL-MSCs were incubated with IMDM containing 50 *μ*mol luciferin-IPA, at 37°C. Three hours later, 50 *μ*L of medium was transferred in a 96-well opaque white plate (Costar), mixed with 50 *μ*L of luciferin detection reagent and incubated for 20 min at room temperature. Luminescence was measured using a Victor3 luminometer.

### 2.10. Quantitative Real-Time Reverse Transcription-Polymerase Chain Reaction

At the end of each step of the differentiation process, both undifferentiated and differentiated FL-MSCs were recovered for total RNA extraction using the TriPure isolation reagent (Roche). After extraction, RNA was quantified using a NanoDrop (Thermo Scientific, Waltham, MA, USA). First-strand cDNA was synthesized using a superscript II cDNA synthesis kit according to the manufacturer's instructions (Invitrogen) and subsequently diluted with nuclease-free water (Invitrogen) to 10 ng/mL cDNA. PCR amplification mixtures (25 *μ*L) containing 25 ng template cDNA, Master Mix buffer (12.5 *μ*L; Applied Biosystems), and 300 nM forward and reverse primer PCRs were run in duplicate and performed on a StepOnePlus Real-Time PCR (Applied Biosystems). The cycling conditions comprised 10 min polymerase activation at 95°C and 40 cycles at 95°C for 15 s and 60°C for 20 s and 72°C for 10 min. Each PCR was followed by a melting curve analysis. Relative quantification was normalized against the house keeping gene GAPDH and/or PPIA. The AB primers and probes used for the current study are listed in Table 2.

### 2.11. Mice Transplantation

Animal studies of the current work were conducted according to protocols approved by the local ethics committee (C2EA-26: Comité d'Éthique en Expérimentation Animale de l'IRCIV; #509/2015062215414016). Differentiated and nondifferentiated FL-MSCs were detached using trypsin-EDTA solution, centrifuged, washed, and suspended at 10^5^ cells/35 *μ*L of physiological serum. Newborn (2 days) NOD/SCID mice were intrahepatically injected using a 0.5 mL insulin syringe (31G) (*n* = 6 for each group). Eight weeks after transplantation, animals were sacrificed. Livers were harvested and fixed in 4% formaldehyde solution and then embedded in paraffin for immunohistochemistry analyses.

### 2.12. Immunohistochemistry

Five *μ*m thick liver sections of NOD/SCID mice were deparaffinized and rehydrated in graded alcohol; then, endogenous peroxidase activity was blocked by incubation for 15 min in a 3% hydrogen peroxide methanol solution (Prolabo). To retrieve the antigens, liver sections were incubated in citric acid monohydrate solution (Dako) at 97°C for 90 min. To block nonspecific staining, sections were incubated for 1 h in 5% normal goat serum (Sigma) diluted in PBS at room temperature. Slices were incubated overnight with polyclonal anti-human albumin (Calbiochem, UK) and polyclonal anti-human ornithine transcarbamylase (OTC) (Sigma, Belgium) in 2% normal goat serum at 4°C. After incubation of the slices with Envision Dako anti-mouse or anti-rabbit (Dako, Belgium) (Table 1), staining was visualized using the chromogenic substrate diaminobenzidine (Sigma, Belgium). Counterstaining was performed using Mayer's hematoxylin (Sigma-Aldrich) for 5 min and mounted using Neo-Entellan Mounting Medium (Merck) for analysis. Mounted slides were examined using HP50 inverted microscope coupled to a DFC camera (Leica, Switzerland). Digital images were acquired using Leica IM50 Image Manager Software. Liver slice samples of transplanted mice were compared to both human liver and nontransplanted mice.

### 2.13. Statistical Analysis

Results are expressed as mean ± standard error of the mean (SEM). Analyses were done using the GraphPad Prism software program (San Diego, California, USA). Statistical differences were determined by Student's *t*-test for two groups' comparison. Differences were considered significant when *p* values were ^*∗*^ *p* < 0.05, ^*∗∗*^ *p* < 0.01, and ^*∗∗∗*^ *p* < 0.001.

## 3. Results

### 3.1. Characterization of Isolated Naive FL-MSCs

**Figure 1:**
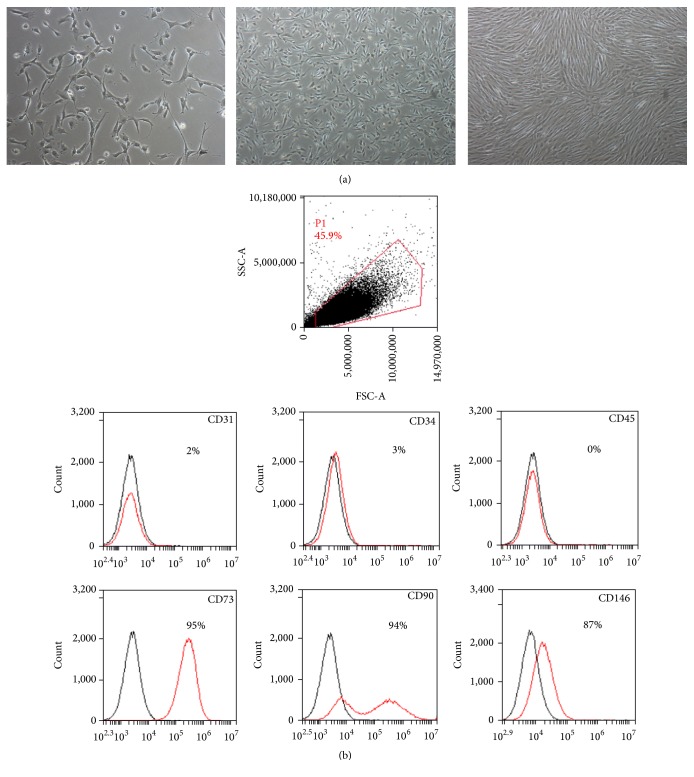
FL-MSC characterization. (a) Morphology of primary layers of adherent MSCs isolated from human fetal liver at low, medium, and high confluence. Magnification: 100x. (b) Cell surface markers expression profile in FL-MSCs. CD31, CD34, CD45, CD73, CD90, and CD146 markers were analyzed using flow cytometry. Immunopositive expression is shown as histograms (red) and compared to corresponding control isotypes IgG (black). The flow cytometry histograms are representative of at least three cell populations isolated from different livers.

**Figure 2:**
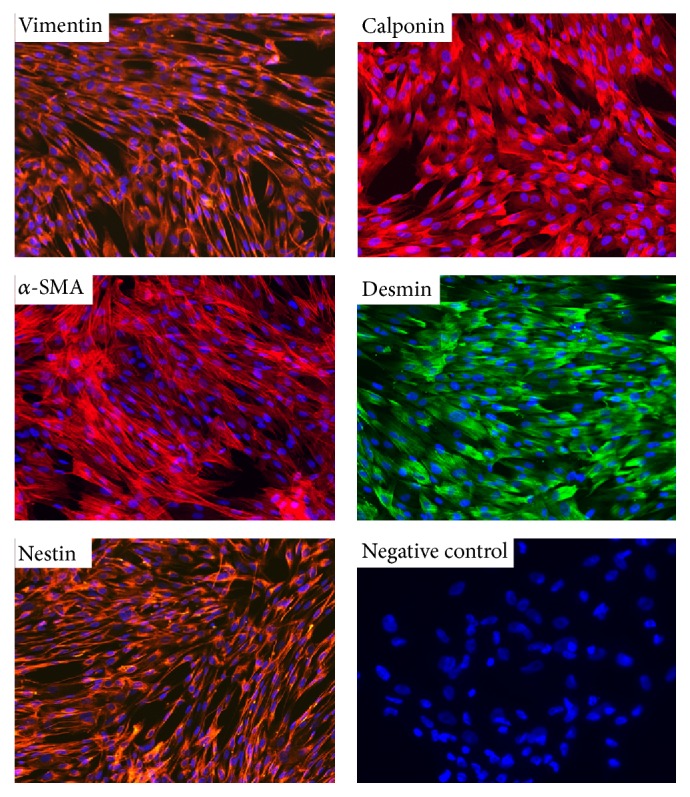
Expression of mesenchymal markers in FL-MSCs. Mesenchymal markers (vimentin and nestin) and smooth muscle markers (calponin, desmin, and *α*-SMA) immunostaining was evaluated using validated corresponding primary antibodies and fluorescence microscopy. Images are representative of several fields examined from the four cell populations. Cell nuclei are stained using DAPI (blue). Negative control: secondary antibody alone. Magnification: 200x.

**Figure 3:**
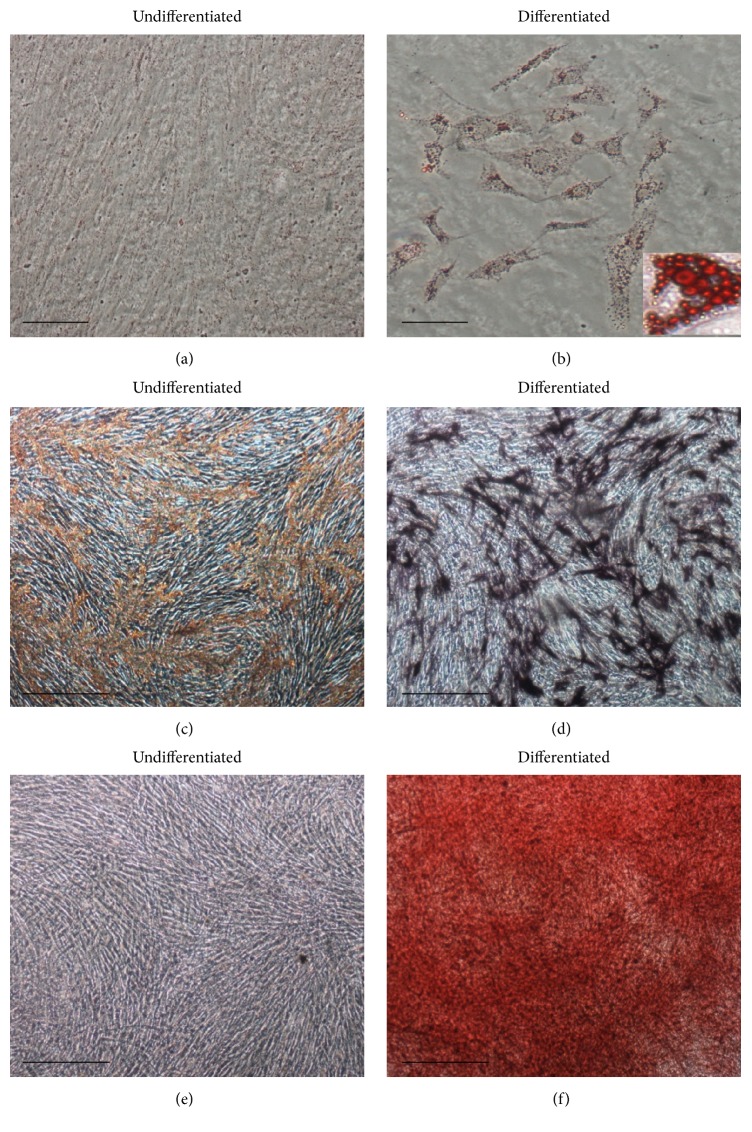
*In vitro* mesodermal differentiation of FL-MSCs. (a, b) Adipogenic differentiation, oil red O staining after 21 days of culture in adipogenic medium (b) or corresponding control cultures (a). Bar = 20 *μ*m. (c, f) Osteogenic differentiation, alkaline phosphatase staining after 6 days of culture in osteogenic medium (d) or corresponding control cultures (c). Alizarin red staining after 21 days of culture in osteogenic medium (f) or corresponding control cultures (e). Bar = 50 *μ*m.

Four different fetal livers (GW 11-12) were digested using collagenase and cells were isolated. For all donors, cell viability estimated by trypan blue exclusion always exceeded 90%. After subsequent plating and first enrichment by plastic adherence, cells with fibroblastic shape started proliferating on the third day after plating and confluence was reached after 7–10 days (Figure 1(a)). While emerging cell populations were heterogeneous before the first detachment, cells presenting mesenchymal morphology became predominant after the first passage (Figure 1(a)). The mesenchymal phenotype of the cells was then demonstrated using flow cytometry which revealed their immunopositivity for CD73, CD90, and CD146 and immunonegativity for hematopoietic CD45 and endothelial CD34 and CD31 markers (Figure 1(b)). Using immunofluorescence, we also showed that the whole FL-MSC population was immunopositive for vimentin and nestin (type III and VI intermediate filaments, resp.), well described in MSC of other sources (Figure 2). We also demonstrated that smooth muscle (SM) cell markers calponin, *α*SM actin, and desmin are positively expressed in all analyzed passages. Upon adipogenic differentiation, FL-MSCs accumulated intracellular lipid droplets as revealed by oil red O staining (Figures 3(a) and 3(b)). Upon osteogenic differentiation, differentiated FL-MSCs showed calcium phosphate precipitates as revealed by alkaline phosphatase activity (Figures 3(c) and 3(d)) and alizarin red staining (Figures 3(e) and 3(f)). All these features confirmed the MSC phenotype of the fibroblastoid cells we isolated from fetal liver as previously reported by our team [8].

### 3.2. *In Vitro* Hepatocytic Differentiation

**Figure 4:**
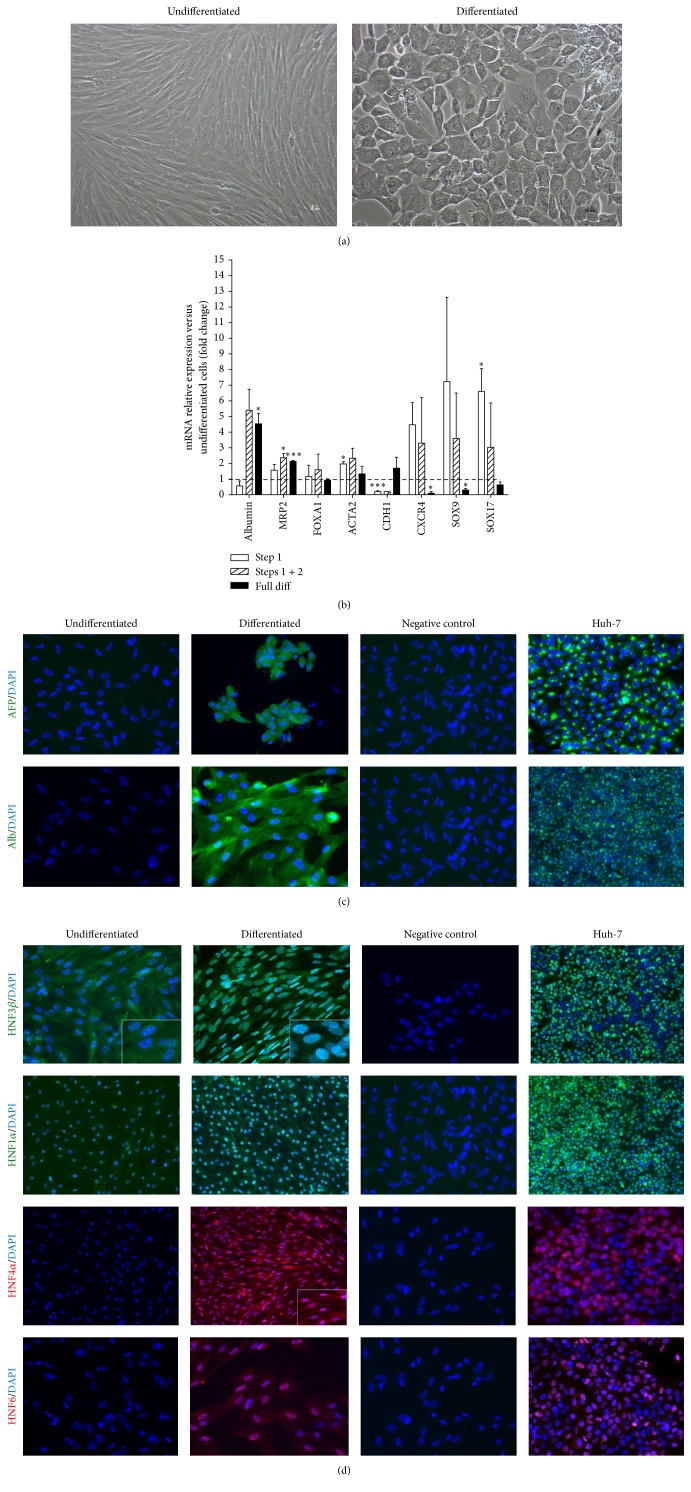
*In vitro* hepatocytic differentiation of FL-MSCs. (a) Morphological changes noticed in differentiated FL-MSCs after 3-4 weeks of culture in hepatogenic differentiation medium as compared to undifferentiated cells. Magnification: 100x. (b) RT-qPCR gene expression analysis demonstrated the decrease in stemness markers and the upregulation of hepatocyte markers mostly at the last maturation step of the differentiation process which is in correlation with morphological changes. Results are expressed as fold change in differentiated versus undifferentiated FL-MSC.* ALB* (albumin encoding gene),* MRP2* (multidrug resistance-associated protein-2 encoding gene),* FOXA1* (Forkhead Box A1 encoding gene),* ACTA2* (*α*-SM-actin encoding gene),* CDH1* (cadherin-1 encoding gene),* CXCR4* (chemokine (C-X-C motif) receptor 4 encoding gene),* SOX9* (SRY-Related HMG-Box 9 encoding gene), and* SOX17* (SRY-Related HMG-Box 17 encoding gene). (c) Immunoreactivity to human hepatic marker and transcription factor specific antibodies: *α*-fetoprotein (AFP), albumin, HNF (hepatocyte nuclear factor) 3*β*, HNF4*α*, HNF6, and HNF1*α*. Presented images are representative of at least 4 different experiments. Negative control: secondary antibody alone. Scale magnification: 400x (except for HNF1*α* (all cells analyzed); Alb and HNF3b in Huh-7: 200x).

Twenty-four hours after seeding, FL-MSCs were sequentially treated with several growth factors/cytokines to induce hepatocytic differentiation as previously documented (see Section 2). After 3-4 weeks in culture, a change in morphology was observed with acquisition of a polygonal, instead of fibroblastoid, shape and increased cytoplasmic granularity (Figure 4(a)). Noteworthy, this change in morphology is very homogeneous throughout the culture whatever the primary cell sample used for the experiment. First, we analyzed using RT-qPCR the kinetic of hepatocytic differentiation by analyzing the mRNA expression levels of stem nad mesenchymal and hepatocytic markers at the end of each step of the differentiation process. The expression of all studied stem and mesenchymal markers (Sox17, sox9, Cxcr4, and *α*-SM actin) was upregulated after the two first steps of the differentiation process (Figure 4(b)). Thereafter, a downregulation was observed at the last maturation phase except for *α*-SM actin for which expression remains at the levels of undifferentiated FL-MSC. For studied hepatic markers albumin, MPR2 and CDH1, if upregulation was noticed at the end of the whole differentiation process for all the markers, their kinetic remains different. MRP2 mRNA expression was upregulated since the first step of the differentiation process; albumin expression upregulation happens when only HGF is added to the cells whereas CDH1 is upregulated only at the last maturation step (Figure 4(b)).

**Figure 5:**
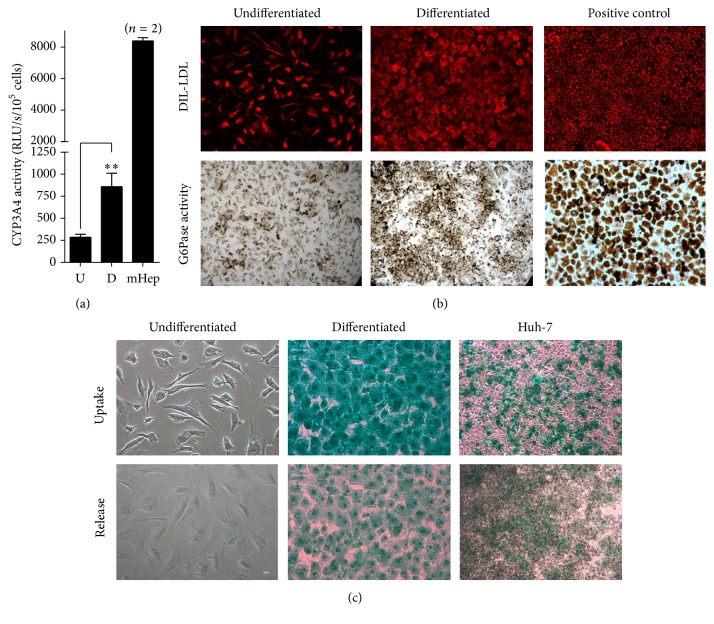
*In vitro* functional hepatocytic differentiation of FL-MSCs. (a) Undifferentiated and differentiated FL-MSCs were incubated with IPA substrate for 4 hours and luciferase activity was measured. Results are expressed as the relative luminescence unit detected in the differentiated (D) cells versus undifferentiated (U) counterparts. Data shown are the mean ± SEM of at least 4 independent experiments. Freshly isolated mouse hepatocytes (*n* = 2), tested under the same experimental conditions for evaluation of CYP3A4 activity, were used as positive controls. (b) Dil-LDL uptake analysis was evaluated after 3 h incubation. Fixed cells were checked using fluorescent microscope. Huh-7 cells were used as a positive control. Pictures were taken at magnification of 200x. The activity of glucose 6-phosphatase (G6Pase) was assessed using cytochemistry. Brown stained cells revealed their ability to convert glucose-6-phosphate substrate to glucose by active G6-Pase. Freshly isolated human hepatocytes were used as a positive control. If both cell groups were displaying Dil-LDL uptake and G6-Pase activity, differentiated FL-MSCs showed more consistent functionalities. All images are representative of 3 different experiments. Magnification: 200x. (c) Indocyanine green uptake and release. Huh-7 cells were used as a positive control. Data shown are representative of 4 different experiments. Magnification: 200x.

Using immunofluorescence, we confirmed the positive protein expression of hepatic markers albumin and *α*-foetoprotein (AFP) in differentiated FL-MSCs (Figure 4(c)). Immunofluorescence studies also indicated major changes in the expression of hepatocyte transcription factors. In differentiated FL-MSCs, HNF4*α* and HNF6 expressions were both induced whereas HNF1*α* and HNF3*β* were enhanced and translocated to the nucleus, respectively (Figure 4(d)). Thereafter, we evaluated the metabolic functionality of the differentiated FL-MSCs as compared to nondifferentiated counterparts. After incubation with an exogenous luciferin-IPA substrate, the intensity of luminescence (reporting the activity of CYP3A4) was significantly induced in differentiated FL-MSCs (~3-fold, *p* < 0.01) (Figure 5(a)). Dil-LDL uptake was detected in undifferentiated FL-MSCs, while it was more enhanced in differentiated cells (Figure 5(b)). Similar results were obtained for glucose-6-phosphatase activity, the final key enzyme of* de novo* glucose production [9], the expression of which was more consistent in differentiated FL-MSCs as compared to undifferentiated cells. In contrast, only differentiated FL-MSCs were able to take up indocyanine green (ICG) after only 1-hour incubation. The differentiated cells also display the potential to release the up taken ICG after 18 h, a function exclusively performed by liver cells (Figure 5(c)).

### 3.3. Cell Engraftment and Hepatocyte Differentiation* In Vivo*

**Figure 6:**
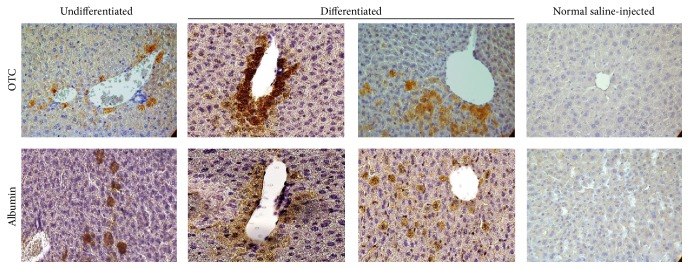
Engraftment and* in situ* differentiation of FL-MSCs in intrahepatically transplanted NOD/SCID mice. OTC (ornithine transcarbamylase, upper panel) and human albumin (lower panel) immunohistochemistry in liver sections from NOD/SCID mice intrahepatically infused with undifferentiated or differentiated FL-MSCs. Analyses were performed at 8 weeks after transplantation and revealed the presence of both integrated scattered cells and clusters of cells expressing both human albumin and OTC, mainly close to vascular structures. Negative control: normal saline-injected mice. Magnification 400x.

To evaluate the FL-MSC potential to be engrafted within a liver, we transplanted newborn NOD-SCID mice intrahepatically using 10^5^ cells, undifferentiated or differentiated. Eight weeks after transplantation, mice were sacrificed and liver sections were analyzed for cell engraftment and* in situ* differentiation. We analyzed, using human-specific antibodies, albumin and ornithine transcarbamylase (OTC) expression to track the injected human cells. We observed, in consecutive liver slices, positive immunostaining for both human albumin and OTC which indicated that cells have differentiated* in situ* (Figure 6). Scattered human cells were noticed in all analyzed mouse livers but most cells were detected as foci next to the portal vascular structures (Figure 6). This parameter has been evaluated using morphometry on scanned slices and by manual counting of the positive cells. Both approaches clearly revealed no differences in the number of positive human cells in both group of cells. Hence, correction of the information has been made in the revised version of the paper.

## 4. Discussion

The current study indicates that MSCs isolated from human 11-12 GW fetal livers can differentiate into hepatocyte-like cells* in vitro* when cultured following a specific protocol and* in vivo* after being intrahepatically transplanted in newborn immune deficient mice.

Plastic adherence and stringent culture conditions have led to selecting nonhematopoietic, spindle shaped, and highly proliferative cells. Even at very low density seeding, nonhematopoietic cells efficiently adhered to the substrate and yielded, after a few passages, a significant population of fibroblastoid cells with no contaminating hematopoietic or hepatoblastic cells. Although, hepatoblast-like cells do form small colonies on the dish, such cell colonies do not proliferate, because culture dishes were not coated with collagen and the culture medium used is not optimal for their culture/proliferation. However, it is difficult to exclude that this small proportion of cells is maintained in the culture and possibly has undergone EMT. Nevertheless, our team has previously demonstrated the high cloning efficiency of the fibroblastoid fetal liver cells on which MSC identity has been confirmed. In that study, we also demonstrated nonsignificant differences between the isolated clones and the primary FL-MSC population in terms of morphology and gene expression.

The isolated fibroblastoid liver cells could be easily expanded up to 15 passages [8]. These cells also fulfilled the other basic criteria for MSC as they exhibited the characteristic surface antigen profile (positivity for CD90, CD73, and CD146 and negativity for CD45, CD31, and CD34) and the adequate intermediate filaments (vimentin and nestin). The typical MSC differentiation potential previously demonstrated for these cells [8] was also shown here for the osteogenic and adipogenic pathways. All these characteristics were shown to be maintained after cryopreservation/thawing. By comparison, multipotency and amplification potential significantly differentiated FL-MSCs from mesenchymal cells obtained from adult human healthy livers [9]. Distinguishing MSC and fibroblasts and thus isolation of the two populations from each other is barely achievable since they share the expression of numerous markers and there is no reliable specific marker expressed by one cell population versus the other one [6].

We then investigated the potential of the FL-MSC populations to differentiate* in vitro* into hepatocyte-like cells following the procedure described for adult human liver mesenchymal stem/progenitor cells [9]. Sequential incubation with specific growth factors/cytokines (FGF2, HGF, and oncostatin M) was applied and several features at the morphological, phenotypic, and functional levels were checked in both differentiated and untreated FL-MSCs. The significant morphological changes observed in all analyzed cell batches of differentiated FL-MSCs were quite comparable to what has been documented for other adult hepatic and extrahepatic mesenchymal cell populations, that is, the polygonal shape and the increased cytoplasmic granularity [10, 11]. Such changes were significantly correlated with an induced hepatogenic mRNA expression profile based on downregulation of stemness markers such as* CXCR4*,* SOX9*,* and SOX17* and upregulation of* ALB* and* MRP2*. The protein expression profile after differentiation showed expression of *α*-fetoprotein and albumin, confirming the upregulation of* ALB* expression. We also observed that the HNF1*α* and HNF3*β* transcription factors, ectopically expressed in undifferentiated FL-MSCs [8], were significantly translocated to the nucleus. Conversely, HNF4*α* and HNF6 that were not expressed before differentiation were induced in hepatogenic conditions. These data show that FL-MSCs display hepatocytic potential as other adult intra- and extrahepatic mesenchymal cells do [9, 10, 12]. The positive expression of hepatocyte nuclear factors before hepatogenic differentiation in FL-MSCs, as well as related high evolving potential towards a hepatic fate as compared to other MSCs of adult origin [8, 13], supported their hepatic origin.

All the above-mentioned features displayed after differentiation led to the investigation of metabolic functionalities. Here, we clearly show that undifferentiated FL-MSCs exhibited both LDL uptake and glucose 6-phosphatase activity and that these metabolic functions were more pronounced after hepatogenic differentiation. On the contrary, uptake/release of indocyanine green was not detected in undifferentiated FL-MSCs while it was displayed after hepatogenic differentiation, indicating their specific ability to display some membrane characteristics of the mature hepatocytes. Likewise, increase in CYP3A4 activity demonstrated the detoxification ability of differentiated FL-MSCs. Altogether these results provide convincing evidence that FL-MSCs can differentiate* in vitro* into cells with significant range of mature hepatocyte functions.

Finally, we investigated the behavior of FL-MSCs* in vivo* after intrahepatic transplantation in newborn NOD-SCID mice. This injection site allows potential availability of attachment factors and extracellular components for transplanted cells to be efficiently delivered and to overcome the acute cell clearance [14]. A mechanical process linked to cell delivery will lead to the entry of the transplanted cells into the vascular spaces. Such ischemic injury by activating many cell types and the secretion of substances will help the permeabilization of endothelial cells. Transplanted cells may then be able to cross the endothelial barrier and to integrate the hepatocytes plates after* in situ* differentiation. Detecting human FL-MSC up to 8 weeks after transplantation suggests their ability to survive and to achieve all these initial steps. This is in accordance with previously published results showing the ability of MSC from different sources to adapt to liver microenvironment [15]. In addition, the immunodetection of human OTC suggests the potential of FL-MSC to differentiate* in situ* and to integrate the hepatocyte plates in the highly proliferative environment characteristic of the newborn liver mice. When undifferentiated or differentiated FL-MSC were transplanted, any differences in terms of engraftment level were noticed as revealed by morphometrical analysis or manual counting of human OTC immunopositive cell number in the recipient mouse livers (data not shown).

## 5. Conclusion

In conclusion, we show here that MSCs generated from 11-12 GW human fetal livers exhibit significant ability to differentiate into hepatocyte-like cells both* in vitro* and* in vivo*. Hence, FL-MSCs are interesting candidates for the investigation of liver development as well as for future cell-based therapy once standardization of the procedure to isolate and expand them will be achieved.

## Figures and Tables

**Table 1:** Antibodies used in this study.

Antibodies/technique used	Supplier	Reference number
*Primary*		
FACS		
CD31	Beckman coulter	IM2409
CD34	Beckman coulter	A07776
CD45	Beckman coulter	A07783
CD73	BD pharmingen	550257
CD146	Biocytex	5050-F100T
Immunofluorescence		
Vimentin	Sigma	V5255
Calponin	Sigma	C2687
*α*-SMA	Sigma	C6198
Nestin	Abcys	VMA5326
Desmin	Sigma	D1033
HNF6	Santa Cruz	sc-13050
HNF4*α*	Santa Cruz	sc-8987
HNF3*β*	Santa Cruz	sc-6554
HNF1*α*	Santa Cruz	sc-135939
*α*-foetoprotein	Santa Cruz	sc-8399
Albumin	Cedarlane	CL2513A
Immunohistochemistry		
Albumin	Calbiochem	126584
OTC	Sigma	HPA000243
*Secondary*		
Donkey anti-rabbit Alexa Fluor 488	Invitrogen	A21206
Donkey anti-rabbit Alexa Fluor 568	Invitrogen	A10042
Donkey anti-goat Alexa Fluor 488	Invitrogen	A11055
Donkey anti-mouse Alexa Fluor 488	Invitrogen	A21202
Donkey anti-mouse Alexa Fluor 568	Invitrogen	A10037
Anti-rabbit (HRP)	Dako	K4003

**Table 2:** Primers used for FL-MSC mRNA analysis.

Gene name	Reference
*ALB*	Hs00910225_m1
*MRP2*	Hs00166123_m1
*FOXA1*	Hs00270129_m1
*ACTA2*	Hs00909449_m1
*CDH1*	Hs01023894_m1
*CXCR4*	Hs00607978_s1
*SOX9*	Hs00165814_m1
*SOX17*	Hs00751752_s1
